# How cultural factors affect medical students' interactions with clinical practice feedback: a qualitative study of ethnically diverse students at three transcontinental campuses

**DOI:** 10.1080/10872981.2025.2567075

**Published:** 2025-10-29

**Authors:** Muirne Spooner, Ciarán Reinhardt, Judith Strawbridge, James Larkin, Siaw Cheok Liew, Mohamed Hasif Jafar, Teresa Pawlikowska

**Affiliations:** a Health Professions Education Centre, Royal College of Surgeons in Ireland University of Medicine and Health Sciences, Dublin, Ireland; b School of Pharmacy, Royal College of Surgeons in Ireland University of Medicine and Health Sciences, Dublin, Ireland; c Department of General Practice, Royal College of Surgeons in Ireland University of Medicine and Health Sciences, Dublin, Ireland; d Perdana University Serdang, Selangor

**Keywords:** Feedback, learning culture, cultural diversity, international medical graduates, feedback literacy

## Abstract

Transnational medical educational programs are now commonplace. Given the importance of individualised feedback, this study explored how cultural factors influence feedback experiences across three transnational campuses of one medical school. A total of 57 final-year medical students were interviewed from a sampling frame of 514 (269 male, 245 female). One-to-one semi-structured interviews were conducted and analysed using template analysis. Codes were iteratively refined into four themes, which were critically reviewed through the lenses of Hofstede’s cultural dimensions and Figured Worlds. Four themes were identified: (1) early socialisation into feedback shaped by family and schooling; (2) hierarchical learning environments positioning students as passive recipients; (3) strategies to cope with negative feedback; and (4) gendered differences in the interpretation of feedback. National identity played only a minor role, while prior experiences and hierarchical structures were more influential. Female students more often described humiliation and emotional burden, whereas some male students framed negative feedback as a ‘rite of passage.’ Feedback experiences in transnational medical programs appear less determined by national culture than by early life experiences and hierarchical clinical environments. Hofstede’s framework offered limited explanatory value, while Figured Worlds illuminated how identities are negotiated in feedback encounters. To enhance feedback cultures in transnational settings, faculty development should prioritise dialogic approaches, sensitivity to learners’ prior experiences, and awareness of gendered impacts. Institutional change is needed to move beyond transmissive practices and foster learner-centred, inclusive feedback.

## Introduction

Culture is frequently proposed as a key influence in learning. Educational practices are influenced by cultural and ideological values [1,2]. A socio-constructivist interpretation of feedback positions it as a ‘social act’ [3]. Learners’ feedback interactions reflect their beliefs and experiences [4], and feedback messages and responses can vary culturally [5,6].

With over 200 medical schools now delivering international programmes, student populations are diversifying and evolving [7]. However, simply delivering an established (often Western or Global North-designed) programme in a different cultural and geographic context can pose significant challenges [8]. There are cross-cultural differences in perceptions of ‘good’ teachers [9]; international medical students may struggle in differing learning environments [10,11]; cultural and normative distance are recognised challenges for universities opening international branch campuses [12]. There is the further challenge that in curricular design, Western standards may be assumed as an ‘ideal’, with concerns that neo-colonialism dictates educational values [1,13]. Simultaneously, with the non-Western voice being under-represented in health professions education research, there is further risk of accepting the published norm as a global norm in education [14,15].

Even without such challenges, learners are consistently dissatisfied with feedback [16], and while feedback has potential to be the most powerful single influence on achievement [17], there exist a number of enablers and barriers influencing feedback uptake [18–20] which limit this influence. Importantly, feedback does not occur in isolation but is embedded within broader systems of assessment and marking, both of which are shaped by cultural norms and expectations. Assessment practices are not culturally neutral; perceptions of fairness, hierarchy, and authority influence how marking is undertaken and interpreted, and in turn how feedback is received and acted upon [21,22]. Students’ expectations of assessment feedback are also shaped by their prior educational experiences, which vary significantly across cultures. Learners from systems dominated by summative assessment often interpret feedback as judgmental and final, while those accustomed to formative traditions anticipate dialogue and developmental guidance [23]. When these ingrained expectations clash with unfamiliar assessment methodologies, students can experience confusion and tension, hindering learning and adaptation.

Large-scale analyses of postgraduate medical examinations further illustrate how ethnicity and cultural background intersect with assessment outcomes. Differential attainment across exam formats has been reported and may reflects broader, systemic cultural factors in assessment [24,25]. These findings underscore those cultural influences extend beyond feedback encounters to the very structures of marking and assessment. As a result, suboptimal practices within these systems not only risk learner confusion and disengagement, but also perpetuate inequities in performance and progression, thereby influencing how feedback is perceived, valued, and acted upon [26].

It is known that cultural context is important in educational design. It is further known that feedback is a complex, contextualised process that is supported through learner-centred strategies. Although feedback literacy has been widely discussed, much of the literature assumes that it develops in a uniform way for all students [27], rather than recognising that it may emerge differently depending on cultural and contextual factors. Prior work has highlighted the influence of contextual, social, and individual elements [3,28]. However, the development of feedback literacy has not yet been explicitly examined within increasingly internationalised and intercultural higher education systems. There is therefore a gap in understanding of how cultural factors specifically interact with feedback, particularly in relation to undergraduate medical students. Our aim is to develop greater understanding of these cultural factors as they shape feedback experiences. By doing this, we aim to support educators in accounting for them in informed feedback design. Additionally, we aim to highlight the voices of students at non-Western sites and non-Western students at a Western site with regard to their feedback experiences. The objectives were to collect and analyse data on feedback experiences from diverse final-year medical students across three international campuses (Ireland, Bahrain, Malaysia). Our research question – *in what way can cultural factors affect medical students’ clinical feedback experiences?* – has not been previously answered and therefore offers new and novel contributions to the medical education literature.

### Theoretical framework

Sociocultural and situated learning theories suggest that learning occurs through interaction and enculturation [29,30]. Within Communities of Practice, novices progress from peripheral to full participation by observing and engaging with group members. From a socio-constructivist stance, feedback must be understood across multiple cultural lenses – national identity, learning environment, and individual experience. To explore these complexities, we pragmatically draw on *Figured Worlds* theory, which has been applied in health professions education to illuminate learner identity formation.

#### Figured Worlds

Feedback responses are highly contextualised [4,26]. Figured Worlds describes how individuals make sense of their social worlds, navigate power, and construct identities within institutional and cultural contexts [31]. Health professions education has used this framework to examine identity in clinical settings [32,33]. Learners simultaneously inhabit multiple worlds – family, community, hospital ward – with medicine itself representing a ‘landscape of communities’ [34]. These overlapping cultures, shaped by specialty, hierarchy, and geography, carry distinct norms around feedback [35].

Within these worlds, feedback influences professional identity formation (PIF) by shaping self-perception and belonging [36–38]. Identity is ‘figured’ through interactions that define what it means to be a ‘good student’ or ‘respected doctor’ [39,40]. Importantly, learners are active participants: their responses to feedback both reflect and reshape these worlds. We therefore use Figured Worlds to recognise feedback as a culturally mediated practice, central to how students construct professional identities across diverse contexts.

#### Alternative theories

Hofstede’s cultural dimensions (1984) offer a complementary lens to explore national influences on learning [41,42], though their validity in education is contested [43]. Critics argue that Hofstede over-simplifies cultural variability [44,45]. Despite these limitations, the model remains widely applied across the social sciences, demonstrating meaningful relationships between culture and societal practices.

## Methods and methodology

### Study design

Deriving from a socio-constructivist epistemology, we believe participants construct their meaning of feedback influenced by their beliefs and experiences as situated in a specific social and cultural context. The methodological approach is exploratory, using qualitative methods in interpreting medical students’ interactions with feedback as they relate to cultural influences. This study is reported in accordance with criteria for reporting qualitative research (COREQ) [46].

### Setting and participants

The study was undertaken at XXXX, which is is ranked in the top 50 Times Higher Education World University Rankings for ‘International Outlook’, with students from over 101 countries (Reference available in non-anonymised text). The study population was final-year medical students enrolled at one of three campuses (Ireland, Bahrain, and Malaysia). The Irish campus has 80% international students, i.e., they are normally citizens or residents outside Ireland. Of the Bahraini students, 60% live in Middle Eastern countries, while 20% are American or Canadian. The remainder are from European, African and Asian countries. All of the Malaysian students are local citizens. The programme is delivered in English at all sites. Final-year students rotate through medical and surgical specialities, with each student spending four weeks on each of general intern al medicine and general surgery rotations. They also undertake an additional eight weeks of clinical rotations in medicine and surgery sub-specialities, e.g., neurology, nephrology fr medicine, plastics and reconstructive surgery, intensive care for surgery, as some examples. In Malaysia, four weeks of orthopaedics training are mandatory, while most only spend two weeks in this specialty at other sites. In Bahrain, intensive care attachments are not available.

The students in the medical programme in Ireland enter via direct or graduate entry, with direct entry to either a six-year programme including a foundation year (pre-medical course) or immediate commencement in the five-year programme, based on the students’ second-level subject choices. The graduate entry programme is an accelerated four-year course, with the students joining those from both direct entry pathways for the final two clinical years. Feedback in the clinical environment is primarily verbal. Mandatory written end-of-rotation appraisals must be elicited; these are available to the student for their portfolio, but not provided to external bodies, e.g., intern or residency programmes. Students also have mandatory meetings with a staff member several times per year for progress reviews. There are two common supervisors at each site, who give feedback on the wards: consultants (attendings) and clinical lecturers (doctors at residency level employed by the medical school to teach and assess on the wards).

### Reflexivity and rigour

Our research team included medical doctors, educational and healthcare psychologists, an infectious diseases consultant and a recent medical school graduate, working across Ireland, UK, Bahrain and Malaysia. The medical school graduate had completed the graduate entry medicine programme in Ireland. As we iteratively explored the data, we applied crystallisation, introducing additional theories to *a priori* themes to view our data from multiple lenses and angles of approach to allow rich exploration of the complexities of culture in learner feedback experience [47,48]. We felt comfortable challenging each other’s preconceptions in refining and agreeing interpretation after independently reading the data. We undertook member validity in two ways – we showed them original transcripts and our interpretation, and our visual interpretations via the storyboard, (Figure 2), both of which were refined from their input.

### Sample and procedures

A PowerPoint presentation introduced the study and research questions to the participants who were final-year medical students at each site, with recruitment announcements via the virtual learning environment and email. Participant information was provided in hard copy and digitally. M.S. developed the interview guide, based on the research questions and cultural theories, refining after a pilot interview (M.S., J.L., T.P.) (Appendix A). Semi-structured interviews explored students’ feedback experiences in the clinical environment.

### Data collection

Participants provided verbal and written consent. Students provided their nationality and named the ‘Place I Call Home’ (PICH). We employed maximum diversity purposive sampling for gender, site of study and PICH, across three campuses. All student participants had completed the same amount of their clinical rotations at time of interviewing in similar rotations, as outlined above. Most interviews (*n* = 49) were conducted in person, some were by online meetings (*n* = 8). We recruited 57 final-year medical students across the three campuses. Planning of the sample size was guided primarily by the principle of *information power*, which recognises that the adequacy of a sample is not defined by numbers alone but by the richness and relevance of the data in relation to the study aim, participant specificity, theoretical framing, interview quality, and analytic strategy [49]. Our study aim was focused, the sample was specific (final-year students across three culturally distinct campuses), and analysis was theoretically informed, all of which indicated that a moderate sample would provide sufficient information power. To ensure adequacy, the first five interviews were reviewed and coded using template analysis, after which further interviews were undertaken iteratively. Recruitment continued until additional interviews yielded no substantively new insights, consistent with the operational use of saturation [50]. The final sample of 57 participants was therefore judged to be adequate to address the study aims. Interviews ranged from 17 to 66 minutes, and interviews were recorded and transcribed initially using transcription software (Otter.ai), edited for accuracy, and anonymised. Original recordings were destroyed after participants were given an opportunity to review transcripts.

## Data analysis

Data were analysed using Template Analysis [51] a systematic form of thematic analysis that differs from Braun and Clarke’s reflexive approach [52] by explicitly permitting the use of a priori codes. This was appropriate for our study, which was informed by existing literature on feedback responses [26] and theoretical frameworks including Hofstede’s cultural dimensions [53] and Figured Worlds [54].

The initial coding and development of the template were led by MS (first author), a clinician-educator with experience in qualitative methods, and then discussed with J.L., C.R., and T.P., who contributed to refining codes and themes. Analysis began after the first five interviews, which were reviewed and coded to produce a preliminary template. This early analysis allowed emerging insights to inform minor adaptations to the topic guide and to shape the focus of subsequent interviews. Data collection and analysis therefore proceeded iteratively, with the coding template refined in parallel with recruitment and interviewing (Table 1).

**Table 1.** Hofstede’s cultural dimensions and dimension ratings for countries at which campuses are located, derived from https://www.hofstede-insights.com/country-comparison-tool?countries=ireland%2cBahrain*%2cMalaysia.

		Ireland	Bahrain	Malaysia
Power distance	The extent to which the less powerful members of institutions and organisations within a country expect and accept that power is distributed unequally.	Low	High	High
Individualism	The degree of interdependence a society maintains among its members. It has to do with whether people’s self-image is defined in terms of ‘I’ or ‘We’.	Individualist	Collectivist	Collectivist
Masculinity/ femininity	Masculine society-driven by competition, achievement and success, with success being defined by the winner/best in field. Feminine society-dominant values in society are caring for others and quality of life. A Feminine society is one where quality of life is the sign of success and standing out from the crowd is not admirable.	Masculine	Masculine	Equal
Uncertainty avoidance	The extent to which the members of a culture feel threatened by ambiguous or unknown situations and have created beliefs and institutions that try to avoid these.	Low	High	Low
Long-term orientation	Normative-prefer to maintain time-honoured traditions and norms while viewing societal change with suspicion. Pragmatic-encourage thrift and efforts in modern education as a way to prepare for the future.	Normative	Not on record	Normative
Indulgence	Indulgent indicates society that prioritises enjoying life and leisure time. Restrained societies prioritise work and minimise importance of leisure time.	Indulgent	Not on record	Indulgent

The process followed King's six stages: familiarisation, preliminary coding, clustering, development of an initial coding template, iterative refinement, and final interpretation. This approach enabled us to combine theoretical concepts with inductive insights. Once all 57 interviews had been coded, the template was stabilised and themes were mapped to the theoretical frameworks. The evolution of the coding template and its links to Hofstede and Figured Worlds are summarised in Tables 2 and 3.

**Table 2.** Component of *Figured World*s used in analysis. Becoming a learner was shaped by culturally valued ways of knowing and acting peculiar to the cultural world of the clinical feedback world. The core practice within a feedback scenario was listening to the supervisor. Students rarely considered it a dialogue, or opportunity for discussion. Conversation centred on identifying and correcting weaknesses. Artefacts included supervisors referring to and demonstrating skills techniques themselves, or verbally representing descriptions of the ideal performance and standard.

*Figured World* feature	Definition	Meaning as identified in interviews
Meaningful acts	Self-evident behaviours, rituals and events	Ward based feedback- on ward rounds, in tutorials Feedback on formative and summative assessments End-of-rotation appraisals
Figures	Persons fulfilling prototypical roles	The stern supervisor The collaborative junior faculty member The student peer
Artefacts	Regularly encountered resources with cultural meaning	Cognitive representations of the ‘ideal standard’ Techniques for performing skills
Figured language	Typical linguistic and narrative strategies	Feedback sandwiches Didactic monologue Castigate, remonstrate Bidirectional dialogue (uncommon) Commiserative (with student peers)

**Table 3.** Evolution of template (high-levels only).

	First iteration	Second iteration	Third iteration	Fourth iteration	Fifth iteration	Sixth iteration
Actions taken		Research group members coding of interviews	Multiple rounds of coding by MS/TP independently and then together			
Description	Derived from Hofstede and literature review	Emphasis on identifying broad feedback themes impacted by socio-cultural factors	Separation based on personal, inter-personal and environmental	Separation focussing on learner-centred factors	Alignment with cultural factors and responses	Alignment with cultural theories
Themes and hierarchical structure	Individualism/collectivismuncertainty toleranceindulgence/restraintmasculinity/femininitylong/short term orientationpower distancerelationshipscharacterssocial context	**Feedback content** Content- level of consistency Content –level of instruction Content –level of clarity Content- criticism/correction focus **Feedback delivery** Depends on supervisor Monologue/Dialogue Non-verbal indicators **Experiences** Positive Emotion-neutral Supportive supervisor Task/behaviour focus Examples Action plans Negative Emotion-centred Stern, harsh supervisor Character/person- focus	**Personal** Conception of FB Preferences in FB Life experiences with FB **Inter-personal** Relationships Power distance Working together Venting/Emotional support **Environment** Wards versus classroom versus lectures Individual versus group Clinicians versus faculty	**Characters** Supervisors Parents Peers Non-medical teachers Ethnic/national background **Life experiences** Childhood/Early life Academic Non-academic **Learner Behaviours** Clarifying messages Challenging messages Performative agreement Performative enacting Changes in learning strategies	**Environment** Access to feedback Feedback content Feedback delivery Public/group feedback Upbringing **Relationships** Uni-directional/transmissive Collaborative Making sense together Commiserating/emotional support Developing preferences **Learner responses** Normalising negative emotions Enculturation Performative adaptation Communities of Practice Withdrawal	**Learning cultures** ***Early-life*** Parents Teachers Coaches ***Clinical medicine*** Hierarchical Hostile Low access Burdensome Contradictory ***Learning culture charactersconsultant supervisors*** Submissive recipients Stoic accepters Performative appeasers ***Faculty supervisors*** Peer collaborator ***Student colleagues*** Battle buddies **National identity** Comforting familiarity Superficial link

## Results

### Participant characteristics

A total of 57 student interviews were conducted at the three sites (26 male students (46%), 31 female students (54%). In the full class, 269 students (52%) were male and 245 students (48%) were female. Regarding entry pathway, 47 students (82%) were enrolled in the Direct Entry programme, while 10 students (18%) were in the Graduate Entry programme, compared with full class of 449 students (87%) in Direct Entry and 65 (13%) in Graduate Entry. Average student age was 25.1 years, with ages ranging from 21 to 33. When asked about the place they call home, 8 students (14.0%) identified Europe, 20 students (35%) identified the Arab States, 14 students (25%) were from the US and Canada, 9 students (16%) were from South East Asia, and 6 students (11%) identified with another region. In the full class, 87 students (17%) called Europe home, 227 students (44%) were from the Arab States, 100 students (20%) were from the US and Canada, 69 students (13%) were from South East Asia, and 31 students (6%) identified with another region.

The participants describe two socially constructed worlds impacting their feedback experiences:

A. Feedback and early-life experience
B. Clinical learning environment

#### *Figured* *World* of early life experience

### Socialisation and early-life contexts

Most students emphasised the formative role of family and early coaching experiences in shaping their orientations to feedback. These early experiences acted as formative Figured Worlds, establishing norms around authority and discipline that foreshadowed later experiences of power play in medicine.

One student indicates they were accustomed to ‘harsh’ feedback, normalising a more combative feedback style:

*‘I think I take feedback really well, especially harsh feedback, because my parents have not been… they have no problem screaming, shouting “that's not enough” kind of thing.’* (S38, male, PICH: Ireland, Ethnicity: Asian Irish, Campus: Ireland)

Another student was averse to ‘harshness’ because their mother showed ‘how good constructive criticism can be delivered from, a very early age’ (S36, female, PICH: Ireland, Ethnicity: White Irish, Campus: Ireland).

Learners describe coaching experiences as formative in their feedback preferences. Indeed, they indicate that these experiences left them more or less equipped to process feedback in Clinical Medicine environments, which they described as homogenous, stylistically criticism-focussed.

*‘I’m used to that more kind of combative, (feedback) might be the right word… when you were training…someone would kinda shout at you, give out…so I suppose it's probably just if you're used to it, it might not be as bad.’* (S37,male, PICH: Ireland, Ethnicity: White Irish, Campus: Ireland)

### Identity and variation

**Figure 1.**
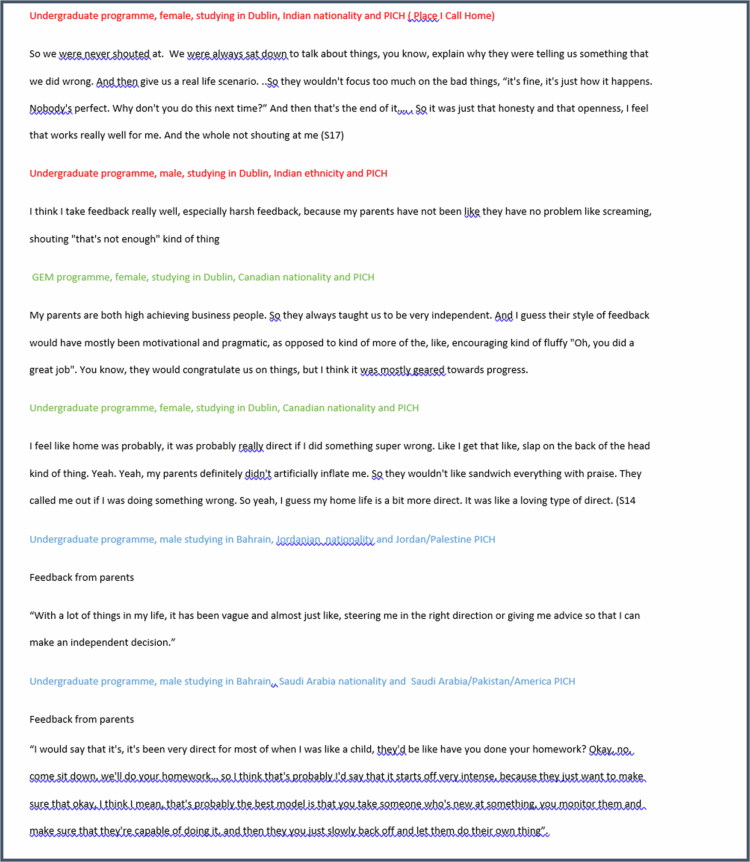
Sample cases of students with similar background and differing life experiences influencing feedback preferences.

Despite shared broader contexts (e.g., national identity or campus location), individual experiences create diverse *Figured World*s, leading to differing responses to feedback (Figure 1). So while there was a prevailing perspective that their responses to feedback are deeply rooted in the social and cultural contexts of their early-life experiences, there was divergence in these experiences which cut across nationality, ethnicity, gender and campus, with students of similar background in these terms describing markedly different orientations.

Examples in Figure 1 show contrasting experiences at the same campus. The contrasting views highlight the *Figured World* concept of variation in shared spaces- overlap of national identity and campus site does not homogenise experiences because individual *Figured World*s (e.g., parental style, coaching experiences) hold greater influence in shaping feedback orientations.

## *Figured World* of clinical medicine

The dominant experience was of criticism-focused feedback as a global medical norm. Students across all campuses and regions described medicine as a profession characterised by harsh criticism and hierarchical feedback, often framed as a ‘rite of passage.’ The medical profession is depicted as a distinct *Figured World* characterised by hierarchical structures and a criticism-focused approach to feedback, shaping students' identities and interactions within its norms.

### Criticism-focussed

Participants describe harsh criticism as a ceremonial norm, likening it to a ‘rite of passage’ necessary for membership within the medical community. This mirrors the notion of *cultural scripts* in *Figured Worlds*, where rituals and shared practices sustain the culture- scripts, or guidelines for the enactment of the various figures of a specific world.

*‘It’s like a rite of passage. I don't think it's just in Dublin, even back home (Caribbean), we would have doctors…there are just some consultants- I think it's just medicine. And I did my electives in North America. Everywhere.’* (S15, female, PICH: Trinidad, Ethnicity: Black Caribbean, Campus: Ireland)

A Malaysian student in Dublin said her friends at home had similar experiences;

*‘when I asked my friends who study medicine in Malaysia- the professors are very, like, rude…they (students at this school) get anxiety as well.*’ (S9, female, PICH: Malaysia, Ethnicity: Asian, Campus: Ireland)

Reports were strikingly consistent across continents, suggesting a transnational figured world of medicine: with shared rituals of criticism and hierarchical interactions.

Ward-based activities meant feedback was often public and students contended with ‘embarrassing’ experiences and ‘harsh’ supervisors. A participant described a typical witnessed encounter:

‘*A big doctor in the college just screamed, was not happy with her answer and told her “you HAVE to know this for the exam”, like, he doesn't say it twice. He's like, “you have to know” Stern.’* (S38, male, PICH: Ireland, Ethnicity: Asian Irish, Campus: Ireland)

Despite this consensus, some students highlighted exceptions when supervisors modelled supportive practices

‘*She was a very nice consultant. A very caring one. And I remember this when she was explaining about the case. She said something that like, it'll remain with me to the last day of my life.’* (S40, male, PICH: Kuwait, Ethnicity: Arabic, Campus: Ireland)

Gender also shaped accounts, with female students more likely to describe harsh public criticism as ‘embarrassing’ and tied to anxiety.

### Hierarchy and positionality

While generally, Hofstede's dimensions [53] did not feature in learners' experiences, participants did highlight power distance, where

*‘there's a clear mentality of “I am your boss and if you talk back to me, I consider”…they would consider that rudeness on the student's part.’* (S12, male, PICH: Kuwait, Ethnicity: Arabic, Campus: Dublin)

Feedback is transmissive due to this power distance with students reluctant to discuss or challenge comments, feeling there is no scope for dialogue.

*‘If it's someone who is higher up the ranks than me, then I mean, medicine is really great for teaching us to keep your mouth shut.”* (S8, female, PICH: Canada, Ethnicity: Asian Canadian, Campus: Ireland)

This aligns with *Figured World*s principles where individuals' roles and agency are constrained by the social hierarchy.

### Burden and accessibility

The *Figured World* of Clinical Medicine also imposes burdens on students due to systemic challenges like resource constraints and overworked staff. Students indicate that

‘*we oftentimes find ourselves getting scolded for asking for feedback.’* (S45, female, PICH: Malaysia, Ethnicity: Asian, Campus: Malaysia)

They understand that staff are under-resourced and this makes accessing feedback challenging. They cannot direct feedback towards their needs because

*‘most of the time, it's kind of like you get what you can get, you know, there's no real option.’* (S50, male, PICH: Qatar, Ethnicity: Arabic, Campus: Bahrain)

Students' experiences suggest that medical education exists at the intersection of multiple figured worlds (e.g., education, healthcare systems), compounding their challenges.

### Learner identify formation and social positioning

Students narrated feedback encounters as identity work within the figured world of medicine, where hierarchy and criticism operate as cultural scripts. They described reading supervisor cues

*‘trying to make fairly snap judgments on who's going to be approachable.’* (S37, male, PICH: Ireland, Ethnicity: White Irish, Campus: Ireland)

They then position themselves into different roles dependent on context.

### Submissive recipient

With senior consultants, students positioned themselves as passive actors, reproducing hierarchy

‘*because considering the hierarchy here, you just smile and nod, basically.’* (S12, male, PICH: Kuwait, Ethnicity: Arabic, Campus: Ireland)

Students feel it is not in their interests to mount resistance to this ‘taken-for-granted’ status quo [55].

### Stoic accepter

When confronted with hostility, some normalised harshness as part of belonging to medicine:

*‘You sort of learn to deal with it. Like it's, it's no longer embarrassing for me like if someone shouted at me, like its fine. And I don't take it to heart at all anymore…whereas in third year, I would have cried, I would have just straight up cried.’* (S17, female, PICH: Tanzania, Ethnicity: Asian, Campus: Ireland)

There is a multi-vocality in their figuring of these supervisors. They recognise the experience of senior supervisors is valuable in providing feedback:

‘*It's cool when you get a consultant to give you feedback on something specific, or someone who's like high status in college. Because you know that they know their things well.’* (S19, male, PICH: Tobago, Ethnicity: Black Caribbean, Campus: Ireland)

This is tempered by fear:

*‘a bad experience from consultants will put me off and I'll be more scared to get more feedback from consultants cos they're mean.”* (S19, male, PICH: Tobago, Ethnicity: Black Caribbean, Campus: Ireland)

### Performative appeaser

Students described ‘playing the game,’ adapting to supervisors’ rigid expectations. Learners did this for ‘show’ and not long-term implementation…Learners take a resigned tone, applying this feedback without any earnestness. Throughout the discourse, feedback activities are figured as something they survive through shrewd analysis of the supervisor’s agenda and playing towards that.

*‘So you then, you try to tailor it to whoever is going to examine you; say, Professor X likes to have us examine a certain way, Professor B has us examine a certain way…I try to bring the things they like, because I know if I don't, even if I was doing everything right, but it's not their way, they will still be like, that's not how you do it.’* (S10, male, PICH: Saudi Arabia, Ethnicity: Arabic, Campus: Ireland)

Learners did this for ‘show’ and not long-term implementation

*‘So I just changed it for that one month I was there and then switch back to the normal way.’* (S6, female, PICH: Canadian, Ethnicity: White Canadian, Campus: Ireland)

Within all these personas, there is cultural reproduction, i.e., the students' acceptance of criticism-focused feedback as a norm, and their perceived need to adapt their identity accordingly, perpetuates the values of this figured world.

### Peer collaborator

In contrast, approachable juniors and faculty enabled dialogic worlds, where learners could co-construct meaning

‘*have been where I was, closer, so they understand, like where I'm coming from.*’ (S11, female, PICH: America, Ethnicity: White American, Campus: Ireland)

This is bi-directional because ‘it's more like informal, and you can have a chat with them *(faculty lecturers).*’ (S10, male, PICH: Saudi Arabia, Ethnicity: Arabic, Campus: Ireland)

*‘Oh, I probably say, “Oh, I heard this from this resource. What are your opinions on that?” So I wouldn't say that to a consultant, I would probably say that to an intern or an SHO.’* (S14, female, PICH: Canada, Ethnicity: White Canadian, Campus: Ireland)

In a discourse centred on concepts of hierarchy and supervisor-centred feedback, these interactions are contrasted for their informality and dialogical nature.

### National and ethnic identity

National identity was less influential than relational dynamics. Some students felt initial affinity with shared backgrounds relating to the *Figured World* concept of social connectedness.

*‘there is kind of just a tendency to, naturally just gravitate towards people who are kind of more…more similar to you.’* (S37, male, PICH: Ireland, Ethnicity: White Irish, Campus: Ireland)

Others emphasised that feedback quality outweighed shared identity.

*‘ If I saw, you know, someone that is Italian, and white and from Canada, I would probably be like, “wow, I'm also Italian, I'm white, from Canada!” But then, you know, when you get to talk to him and see he's also, you know, just completely different from me in every single way ……the most recent experience of a doctor I had, who, who gave me the best feedback ever, and made me want to almost change my, like, my specialty into like, cardiology was, she's a female, Arabic doctor, Muslim, completely different everything, right.’* (S50, male, PICH: Qatar, Ethnicity: Arabic, Campus: Bahrain)

Students' notion of supervisors is figured more on their individual preferences rather than any ethnic or national-derived characteristics. While local context and personal identity formation now intersect, national identity is less intertwined:

*‘I think over time you adjust to the cultures of people who are there (in Medicine) and then you understand how they want things to be done. So I don't think it (nationality/ethnicity) matters in the end.’* (S27, female, PICH: United Arab Emirates, ethnicity: Asian Indian, Campus: Ireland)

### Battle buddies

Peers were authored as ‘battle buddies,’ offering solidarity in this hierarchical world:

*‘Someone who yelled at all the other students, we can kind of joke about it after, it’s like, “oh, he said that same thing to me”.’* (S6, female, PICH: Canada, Ethnicity: White Canadian, Campus: Ireland)

There is a camaraderie in sharing *‘gallows humour’.* They ask them for second opinions when they doubt feedback credibility.

*‘If it's bad feedback, sometimes I have to sort of vent it out and talk to my friends about it, like, oh, “did I really do that poorly?”, or this or that, and it helps me get through it. You know, it also helps me come to the fact that, “Hey, maybe I did do it very poorly.”* (S50, male, PICH: Qatar, Ethnicity: Arabic, Campus: Bahrain)

Peers acted as allies in hostile figured worlds of medicine, offering emotional support and validation that mitigated the negative impact of hierarchical, critical feedback cultures. Taken together, these positional identities indicate students navigate cultural scripts of medicine. By adopting submissive, stoic, or performative roles, they reproduce hierarchical norms, while collaboration with peers and approachable supervisors created alternative figured worlds that resisted dominant patterns of power.

## Discussion

We sought to explore the role of cultural factors in medical students' feedback experiences, and how these affect their response to feedback. To align with our aim of examining factors at individual, environmental, and societal levels, we interpret our findings primarily through the Figured Worlds framework, complemented by Hofstede's dimensions, with Figured Worlds emerging as the more dominant lens.

Across three culturally distinct sites, learners described experiences marked by hierarchy, criticism, and limited access. These shaped a distinct context for learning with specific norms and expectations, i.e., a learning culture [35]. Students have modest expectations of medical teachers' feedback due to a pedagogy of ‘learning by doing’ and self-directedness, as previously reported [56]. Our findings support previous work that feedback experiences are impacted by the perceived learning culture in which they occur [57–59]. Hierarchy culture like our participants experienced, breeds negative experiences [57] and is a barrier to bi-directional feedback [59]. Hierarchy limits agency, and agency itself is conceptualised as a counter-cultural act [60], but it is also a critical characteristic of the proactive learner who seeks out and engages in the necessary back-and-forth to make feedback developmentally meaningful, showcasing an inherent tension for the learner. Our described learning culture contrasts with those of residents who were challenged by a ‘prevailing culture of niceness’ as a barrier to honest feedback [58]. This difference may be somewhat explained due to the nature of the relationship with residents as longer-term colleagues. Positive feedback experiences occur for medical students in the context of trusted relationships in longitudinal clerkships [61], underlining the importance of long-term relationship building for feedback. Therefore, medical schools should prioritise longitudinal placements and continuity of supervision where possible as these conditions foster the trust needed for constructive feedback to flourish.

While students felt their national or ethnic background had little impact, the relationships in their upbringing also significantly influenced their feedback preferences. Parents and coaches represented formative cultures of feedback, aligning with Vygotsky's concept of ‘more knowledgeable others’ in the zone of proximal development (Vygotsky, 1978). Watling describes how learners who had experienced coached disciplines became accustomed to critical feedback [35]; our work adds that learners normalise feedback specifically in the style modelled by early influencers. Models like R2C2 emphasise relationship-building [62]; our findings suggest that part of this feedback relationship should explore learners' early feedback experiences to better align expectations.

Building on how influential actors influence learners, we developed understanding of how students construct their identities and behaviours with specific types of teachers in their clinical workplace [63]. Our participants considered their role in terms of positionality to other actors, with identity formed as a function of cultural experience [31].

Senior clinical staff are ‘stern’ characters who students are reluctant to challenge. Learners self-author as *submissive recipients* in the ‘rite of passage’ of scaling the pecking order. Participants are *stoic acceptors* of humiliating feedback, echoing descriptions of maladaptive response to student mistreatment [64]. Students are performative appeasers – they align values with superiors and perform according to supervisor preference [64,65]. Students adapted to the demands and norms of their educational context [66], accepting criticism-heavy, infrequent feedback as a signature pedagogy of Medicine [67]. These findings reinforce that feedback is not only an instructional exchange but also a cultural practice that shapes professional identity [36,68]. This adaptability risks normalising outdated practices, reinforcing transmissive roles of ‘provider’ and ‘receiver’ [69]. Learners may in fact avoid feedback to protect themselves from harm [26,70], rather than challenging the status quo, further concealing the problem. Our work furthers descriptions of learners who are often ‘striving to survive’, mirroring identities depicted in clinical assessments [71]. This underlines the challenge when learning culture embeds rather than resists unsupportive practice. Such adaptability, while resourceful, risks normalising outdated practices; faculty development must therefore challenge transmissive feedback norms and support dialogic, learner-centred approaches instead.

To combat unhelpful feedback experiences, students regularly reflected with other students. Supervisor-facilitated reflection supports feedback uptake [72,73]. Our work suggests there is potential to leverage peer feedback more formally in the curricula to optimise their own adapted practice with faculty support.

While much previous work has considered cultural differences in terms of national and ethnic backgrounds [74], students' feedback experiences were not clearly shaped by nationality. Power Distance was consistently relevant [75], but broader national cultural dimensions did not explain observed feedback behaviours. In a ‘glocalised’ world [76], traditional cultural boundaries may blur. Post-subculture theory suggests identity is increasingly shaped by individual and group interactions rather than national affiliation [77,78], which reflects our experience. We showcase the strength in researching across multiple sites and learner profile to likely reflect genuine global experiences [79]. This indicates that educators should look beyond nationality when considering feedback cultures, focusing instead on local interactions, relational dynamics, and identity work within specific learning environments.

Our findings highlight that meaningful cultural change requires faculty who are skilled in providing in-the-moment, relationship-based feedback [80] and who are committed to fostering a positive learning environment at an institutional level. Embedding structured peer feedback into curricula could therefore provide an immediate, practical step towards counteracting hierarchical cultures and broadening access to meaningful feedback.

## Strengths and limitations

This study included diverse, multinational students from both graduate- and direct-entry pathways, across three culturally distinct sites. This variety, combined with a sizeable qualitative sample of 57 interviews, enhances the transferability of the findings. Our analysis was situated within clearly articulated theoretical frameworks, supporting *crystallisation* [47] by allowing the data to be interpreted from multiple perspectives.

**Figure 2.**
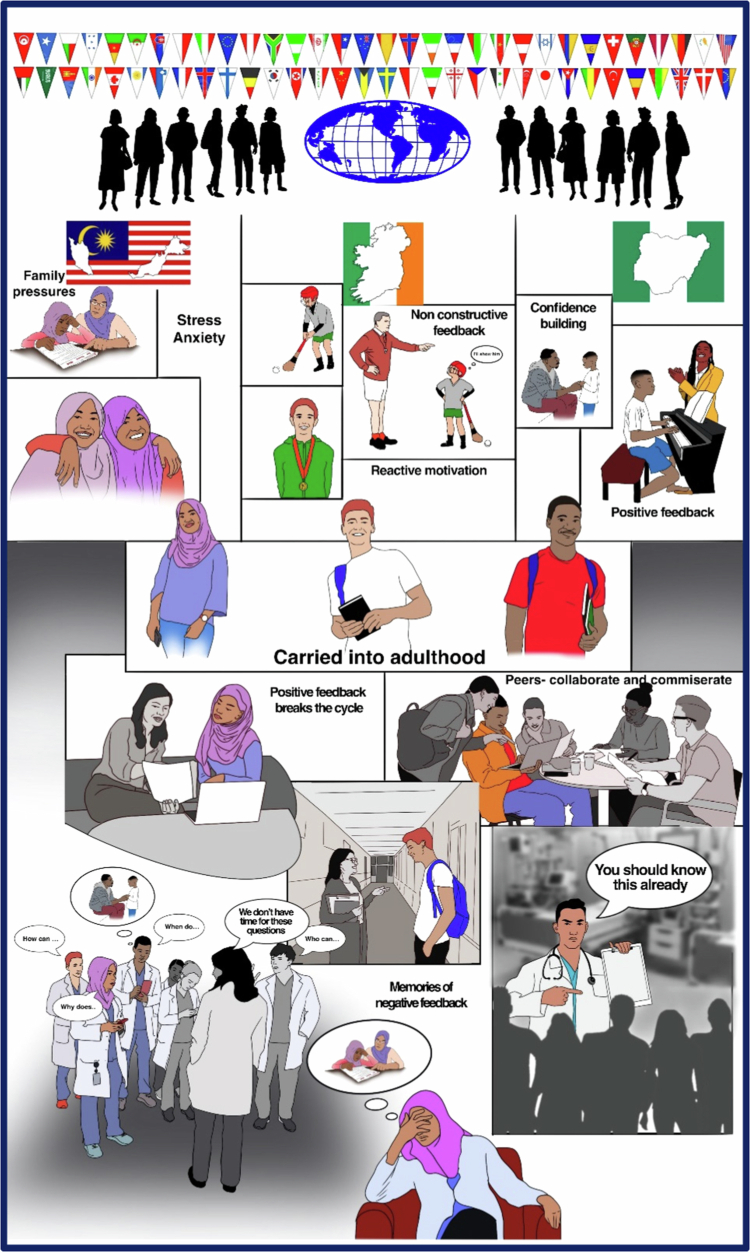
A story-board representation of our students' experiences as developed via-member checking. Students from a diversity of backgrounds come to study at RCSI, Malaysia, Ireland and Bahrain. They bring with them feedback preferences developed in family and early-life teaching experiences. They contend with specific culture in Clinical Medicine with some supportive peer supervisors, a community of practice with peers, and hierarchical feedback experiences that can be hostile and where they feel burdensome, with senior consultant supervisors.

In terms of rigor, member checking was undertaken at two levels: several participants reviewed their transcripts, and a sub-sample also reviewed the emergent themes, which they confirmed reflected their experiences. These steps enhanced the trustworthiness of the analysis. We did not, however, collect field notes or triangulate with other data sources such as supervisor perspectives or student performance measures, which may have provided further depth. Feedback is also a highly individualised experience, and although our sample was large for a qualitative study, it is unlikely to have captured the full range of possible perspectives (Figure 2).

Future research would benefit from incorporating supervisor perspectives, other learner groups, and multiple data sources to further triangulate findings and broaden understanding of how cultural factors shape feedback practices.

## Conclusion

This study demonstrates that students' responses to feedback are shaped not only by national or campus cultures, but also by early-life experiences, local clinical hierarchies, and the relational dynamics of the supervisor–student encounter. The prevailing figured world of medicine is characterised by hierarchical, criticism-focused feedback, yet students adapt in multiple ways – from submissive recipients to collaborative peers – depending on the power relations at play. We highlight the need to create feedback practices that actively reduce unnecessary power distance and foster dialogue. Educators can improve feedback for diverse student groups by:

• modelling constructive, collaborative approaches that signal respect and accessibility;
• recognising that students arrive with varied cultural and early-life orientations to feedback; and
• intentionally providing safe spaces where learners can question, clarify, and co-construct meaning from feedback.

By shifting from criticism-focused rituals to dialogic, learner-centred approaches, educators can better support feedback literacy, professional identity formation, and equitable learning experiences for culturally diverse students.

## Data Availability

The datasets analysed in the study are available from the corresponding author on reasonable request.

## Appendices Appendix A:

### Guiding questions for semi-structured interviews

1. What do you consider to be feedback?
2. Tell me about your feedback experiences before you went to 0I557
3. Are there certain types of feedback that you prefer to others?
4. For you personally, when you seek feedback, what is your main goal?
5. Do you seek feedback? *If yes then move to question 7*
6. If not then why not?
  • Did you have a bad experience with feedback?
  • What would it take for you to seek out feedback?
7. What motivates you to seek feedback? What are your goals when receiving feedback?
  • What motivates you to spend time thinking/engage with the feedback you receive?
  • If you did badly, what would you do?
  • If you disagreed with the feedback, what would you do?
  • How do you decide on who to ask for feedback or to discuss feedback with?
8. Do you ever actively avoid feedback?
  When/what context?
9. How important do you think feedback is in improving your future performance? Can you give an example of where the feedback you got helped you learn and do something better?
10. Does the background of your teacher/supervisor influence whether you’re likely to seek feedback from them? If yes, what characteristics?
  a. How do you see yourself in the relationship with the supervisor?
  b. How do you see the supervisor?
11. Do you consider consistency when asking for feedback?
12. Does feedback change what learning strategies you use? (Learning strategies include using books, seeing patients, practicing exams on friends, etc.) How?
13. Does feedback change how you implement/organise your learning?
14. How do you check if applying feedback has helped your learning?
15. Is there anyone else who is involved with giving you feedback?

Questions are based on Hofstede’s dimensions [74]. A social-cognitive approach to motivation and personality [70] and Identity and Agency in *Figured Worlds* [31], Dweck, C. S., & Leggett, E. L. (1988). A social-cognitive approach to motivation and personality. *Psychological Review, 95* (2), 256. Hofstede, G. (1984). *Culture's consequences: International differences in work-related values* (Vol. 5): Sage. Holland, D., Lachicotte Jr, W., Skinner, D., & Cain, C. (2001). *Identity and agency in cultural worlds*: Harvard University Press.

## Appendix B: Consolidated criteria for reporting qualitative studies (COREQ): 32-item checklist

Developed from:

Tong A, Sainsbury P, Craig J. Consolidated criteria for reporting qualitative research (COREQ): a 32-item checklist for interviews and focus groups. *International Journal for Quality in Health Care*. 2007. Volume 19, Number 6: pp. 349–357.

**Table:**

No. Item	Guide questions/description	Reported on page #
**Domain 1: research team and reflexivity**		
*Personal characteristics*		
1. Inter viewer/facilitator	Which author/s conducted the interview or focus group?	MHJ,JL,MS
2. Credentials	What were the researcher’s credentials? e.g., PhD, MD	MS-MBBCHBAO, PG Dip HPE TP-MB BS MSc PhD MRCP DRCOG FRCPI JL-MHJ- BSC, PhD CSL-MB BS MSc SM-MD, FRCP, FRCPI, AM, DTM&H
3. Occupation	What was their occupation at the time of the study?	MS – senior lecturer TP – Director HPEC JL – doctoral researcher MHJ – Post-doctoral researcher CSL – Associate Professor GP SM – Professor of ID
4. Gender	Was the researcher male or female?	MS – female TP – female JL – male MHJ – male CSL – female SM – male
5. Experience and training	What experience or training did the researcher have?	All experienced qualitative researchers. MS in final stages of PhD and has completed PG Dip in HPE.
*Relationship with participants*		
6. Relationship established	Was a relationship established prior to study commencement?	No, interviewers introduced themselves to participants at time of interviews.
7. Participant knowledge of the interviewer	What did the participants know about the researcher? e.g., personal goals, reasons for doing the research	Participants were briefed on the purpose of the study and understood it. Ethics had granted, participants reviewed the participant information documentation prior to giving their written informed consent to be involved.
8. Interviewer characteristics	What characteristics were reported about the interviewer/facilitator? e.g., Bias, assumptions, reasons and interests in the research topic	MS regularly provides feedback to students, and is completing a PhD on feedback, which is a potential source of bias. No other sources of bias were identified.
**Domain 2: study design**		
*Theoretical framework*		
9. Methodological orientation and Theory	What methodological orientation was stated to underpin the study? e.g., grounded theory, discourse analysis, ethnography, phenomenology, content analysis	Socio-constructivist epistemology, template analysis of interviews, through lens of SRL theory.
*Participant selection*		
10. Sampling	How were participants selected? e.g., purposive, convenience, consecutive, snowball	Purposive
11. Method of approach	How were participants approached? e.g., face-to-face, telephone, mail, email	Virtual learning environment notice and face-to-face communication to explain study. Participants then emailed expression of interest to gatekeeper to arrange interview.
12. Sample size	How many participants were in the study?	57
13. Non-participation	How many people refused to participate or dropped out? Reasons?	None
*Setting*		
14. Setting of data collection	Where was the data collected? e.g., home, clinic, workplace	Meeting room in.HPEC, Dublin. Online for Manama (Bahrain) and Perdana (Malaysia).
15. Presence of non-participants	Was anyone else present besides the participants and researchers?	No
16. Description of sample	What are the important characteristics of the sample? e.g., demographic data, date	Available on request
*Data collection*		
17. Interview guide	Were questions, prompts, guides provided by the authors? Was it pilot tested?	Yes. Interviews were semi-structured using a guide (Appendix A), follow-up questions were allowed. Yes, pilot-testing details.
18. Repeat interviews	Were repeat interviews carried out? If yes, how many?	No
19. Audio/visual recording	Did the research use audio or visual recording to collect the data?	Audio recordings
20. Field notes	Were field notes made during and/or after the interview or focus group?	Yes, brief notes made after interviews to support reflexivity, but not analysed or used for triangulation.
21. Duration	What was the duration of the inter views or focus group?	
22. Data saturation	Was data saturation discussed?	
23. Transcripts returned	Were transcripts returned to participants for comment and/or correction?	No
**Domain 3: analysis and findings**		
*Data analysis*		
24. Number of data coders	How many data coders coded the data?	MS and TP
25. Description of the coding tree	Did authors provide a description of the coding tree?	Table 3
26. Derivation of themes	Were themes identified in advance or derived from the data?	*A priori* themes were developed from SRL theory. Subsequent themes were derived from the data. Themes were compared, edited, deleted when not relevant to the RQ, re-arranged hierarchically.
27. Software	What software, if applicable, was used to manage the data?	Microsoft Word, Excel, N Vivo
28. Participant checking	Did participants provide feedback on the findings?	Yes
*Reporting*		
29. Quotations presented	Were participant quotations presented to illustrate the themes/findings? Was each quotation identified? e.g., participant number	Yes
30. Data and findings consistent	Was there consistency between the data presented and the findings?	Yes
31. Clarity of major themes	Were major themes clearly presented in the findings?	Yes, themes were supported with direct quotes attributed to anonymised participants.
32. Clarity of minor themes	Is there a description of diverse cases or discussion of minor themes?	Yes, minor and diverse themes are discussed in the manuscript.

## Appendix C: Template analysis process

**Table:**

1. Familiarisation with data. 2. MS read through the transcripts at least once and sometimes several times. 3. Preliminary coding. 4. MS undertook initial analysis of a selection of interviews representing each campus site and students of varying PICH and gender. She identified high-level themes and sub-themes relevant to the research questions. 5. Clustering. 6. Newly identified themes were clustered with existing *a priori* codes. 7. Producing an initial template. 8. A draft template was developed from the clustering of high-level themes (MS). 9. Applying and developing the template. 10. As further iterative analysis and discussion ensued (MS, TP), the template was revised to a final agreed hierarchy of major themes and lower-level codes (MS). Analysis was undertaken until it was considered that we had achieved sufficient information power [49]. 11. Template evolution and final interpretation. 12. We initially derived *a priori* codes from literature review. The second iteration of the template iteratively identified three broad themes of feedback impacted by socio-cultural factors. MS and TP used the template to code transcripts to evaluate for themes delete or modify existing themes and change the hierarchy of themes and sub-themes. This led to the development of the third template iteration, focussing on separation in to personal, inter-personal and environmental factors. The final iteration applies three overarching domains, which relate to cultural factors influencing learners’ experiences of, and responses to feedback.

## References

[1] Bleakley A, Brice J, Bligh J. Thinking the post-colonial in medical education. Med Educ. 2008;42(3):266–270. 18275413 10.1111/j.1365-2923.2007.02991.x

[2] Wong AK. Culture in medical education: comparing a Thai and a Canadian residency programme. Med Educ. 2011;45(12):1209–1219. 21999309 10.1111/j.1365-2923.2011.04059.x

[3] Ajjawi R, Boud D. Researching feedback dialogue: an interactional analysis approach. Assess Eval High Educ. 2017;42(2):252–265.

[4] Garino A. Ready, willing and able: a model to explain successful use of feedback. Adv Health Sci Educ. 2020;25(2):337–361. 10.1007/s10459-019-09924-2 31598884

[5] Earley PC, Gibson CB, Chen CC. “How did I do?” versus “How did we do?” Cultural contrasts of performance feedback use and self-efficacy. J Cross-Cult Psychol. 1999;30(5):594–619.

[6] Sully De Luque MF, Sommer SM. The impact of culture on feedback-seeking behavior: an integrated model and propositions. Acad Manage Rev. 2000;25(4):829–849.

[7] Brouwer E, Frambach J, Driessen E. Mapping the scope of internationalized medical education Paper presented at the The Network Towards Unity for Health Annual Conference. 2017.

[8] Frambach JM, Driessen EW, Chan LC, et al. Rethinking the globalisation of problem‐based learning: how culture challenges self‐directed learning. Med Educ. 2012;46(8):738–747. 22803751 10.1111/j.1365-2923.2012.04290.x

[9] Kikukawa M, Nabeta H, Ono M, et al. The characteristics of a good clinical teacher as perceived by resident physicians in Japan: a qualitative study. BMC Med Educ. 2013;13:1–10. 23883367 10.1186/1472-6920-13-100 PMC3728218

[10] Liberman K. Asian student perspectives on American university instruction. Int J Intercult Relat. 1994;18(2):173–192. doi: 10.1016/0147-1767(94)90027-2

[11] Townsend P, Jun Poh H. An exploratory study of international students studying and living in a regional area. J Market High Educ. 2008;18(2):240–263.

[12] Wilkins S. Establishing international branch campuses: a framework for assessing opportunities and risks. J High Educ Policy Manage. 2016;38(2):167–182. doi: 10.1080/1360080X.2016.1150547

[13] Hodges BD, Maniate JM, Martimianakis MA, et al. Cracks and crevices: globalization discourse and medical education. Med Teach. 2009;31(10):910–917. 19877863 10.3109/01421590802534932

[14] Miranda JJ, Zaman MJ. Exporting "failure": why research from rich countries may not benefit the developing world. Rev Saúde Pública. 2010;44:185–189. 20140343 10.1590/s0034-89102010000100020 PMC2871307

[15] Yousif NH, Bonati M. North and South: bridging the information gap. Lancet. 2000;356(9234):1034–1035. 10.1016/S0140-6736(05)72658-1 11041431

[16] England HEFCf. National Student Survey Results 2016. 2016.

[17] Hattie J. Visible learning: a synthesis of over 800 meta-analyses relating to achievement. London: Routledge; 2008.

[18] Carless D, Boud D. The development of student feedback literacy: enabling uptake of feedback. Assess Eval High Educ. 2018;43(8):1315–1325.

[19] Eva KW, Armson H, Holmboe E, et al. Factors influencing responsiveness to feedback: on the interplay between fear, confidence,//reasoning processes. Adv Health Sci Educ. 2012;17(1):15–26. 10.1007/s10459-011-9290-7 PMC3274671 21468778

[20] Winstone NE, Nash RA, Parker M, et al. Supporting learners' agentic engagement with feedback: a systematic review and a taxonomy of recipience processes. Educ Psychol. 2017;52(1):17–37.

[21] Carless D. Differing perceptions in the feedback process. Stud High Educ. 2006;31(2):219–233. doi: 10.1080/03075070600572132

[22] Struyven K, Dochy F, Janssens S. Students’ perceptions about evaluation and assessment in higher education: a review. Assess Eval High Educ. 2005;30(4):325–341.

[23] Rovagnati V, Pitt E. Exploring intercultural dialogic interactions between individuals with diverse feedback literacies. Assess Eval High Educ. 2022;47(7):1057–1070.

[24] McManus I, Elder AT, Dacre J. Investigating possible ethnicity and sex bias in clinical examiners: an analysis of data from the MRCP (UK) PACES and nPACES examinations. BMC Med Educ. 2013;13(1):103. 23899223 10.1186/1472-6920-13-103 PMC3737060

[25] Wakeford R, Denney M, Ludka-Stempien K, et al. Cross-comparison of MRCGP & MRCP (UK) in a database linkage study of 2,284 candidates taking both examinations: assessment of validity and differential performance by ethnicity. BMC Med Educ. 2015;15(1):1. 25592199 10.1186/s12909-014-0281-2 PMC4302509

[26] Spooner M, Duane C, Uygur J, et al. Self–regulatory learning theory as a lens on how undergraduate and postgraduate learners respond to feedback: a BEME scoping review: BEME Guide No. 66. Med Teach. 2022;44(1):3–18. 34666584 10.1080/0142159X.2021.1970732

[27] Pitt E, Bearman M, Esterhazy R. The conundrum of low achievement and feedback for learning. Assess Eval High Educ. 2020.

[28] Chong SW. Reconsidering student feedback literacy from an ecological perspective. Assess Eval High Educ. 2021;46(1):92–104.

[29] Brown JS, Collins A, Duguid P. Situated cognition and the culture of learning. Educ Res. 1989;18(1):32–42.

[30] Lave J, Wenger E. Situated learning: legitimate peripheral participation. Cambridge: Cambridge university press; 1991.

[31] Holland D, Lachicotte, Jr W, Skinner D, et al. Identity and agency in cultural worlds. Cambridge, MA: Harvard University Press; 2001.

[32] Bennett D, Solomon Y, Bergin C, et al. Possibility and agency in Figured Worlds: becoming a ‘good doctor’. Med Educ. 2017;51(3):248–257. 28032364 10.1111/medu.13220

[33] Cantillon P, De Grave W, Dornan T. The social construction of teacher and learner identities in medicine and surgery. Med Educ. 2022;56(6):614–624. 34993973 10.1111/medu.14727 PMC9305233

[34] Wenger-Trayner E, Wenger-Trayner B. Learning in a landscape of practice: a framework. Routledge; 2014. pp. 13–30.

[35] Watling C, Driessen E, van der Vleuten CP, et al. Learning culture and feedback: an international study of medical athletes and musicians. Med Educ. 2014;48(7):713–723. 24909533 10.1111/medu.12407

[36] Dornan T, Pearson E, Carson P, et al. Emotions and identity in the figured world of becoming a doctor. Med Educ. 2015;49(2):174–185. 25626748 10.1111/medu.12587

[37] Mann K, Gordon J, MacLeod A. Reflection and reflective practice in health professions education: a systematic review. Adv Health Sci Educ. 2009;14:595–621. 10.1007/s10459-007-9090-2 18034364

[38] Wong A, Trollope‐Kumar K. Reflections: an inquiry into medical students’ professional identity formation. Med Educ. 2014;48(5):489–501. 24712934 10.1111/medu.12382

[39] Urrieta L. Figured worlds and education: an introduction to the special issue. Urban Rev. 2007;39(2):107–116.

[40] Cruess RL, Cruess SR, Boudreau JD, et al. A schematic representation of the professional identity formation and socialization of medical students and residents: a guide for medical educators. Acad Med. 2015;90(6):718–725. 25785682 10.1097/ACM.0000000000000700

[41] Joy S, Kolb DA. Are there cultural differences in learning style? Int J Intercult Relat. 2009;33(1):69–85.

[42] Monrouxe LV, Chandratilake M, Chen J, et al. Medical students' and trainees' country-by-gender profiles: Hofstede's cultural dimensions across sixteen diverse countries. Frontiers in medicine; 2021;8. 10.3389/fmed.2021.746288 PMC8862177 35211478

[43] Dennehy E. Hofstede and learning in higher level education: an empirical study. Int J Manag Educ. 2015;9(3):323–339.

[44] Signorini P, Wiesemes R, Murphy R. Developing alternative frameworks for exploring intercultural learning: a critique of Hofstede's cultural difference model. Teach High Educ. 2009;14(3):253–264.

[45] Merelman RM. Making something of ourselves. University of California Press; 1984.

[46] Tong A, Sainsbury P, Craig J. Consolidated criteria for reporting qualitative research (COREQ): a 32-item checklist for interviews and focus groups. Int J Qual Health Care. 2007;19(6):349–357. 17872937 10.1093/intqhc/mzm042

[47] Richardson L, St. Pierre EA. Writing: a method of inquiry In: The Sage handbook of qualitative research. 2005. p. 959–968.

[48] Varpio L, Ajjawi R, Monrouxe LV, et al. Shedding the cobra effect: problematising thematic emergence, triangulation, saturation and member checking. Med Educ. 2017;51(1):40–50. 27981658 10.1111/medu.13124

[49] Malterud K, Siersma VD, Guassora AD. Sample size in qualitative interview studies: guided by information power. Qualit Health Res. 2016;26(13):1753–1760. 10.1177/1049732315617444 26613970

[50] Guest G, Bunce A, Johnson L. How many interviews are enough? An experiment with data saturation and variability. Field Methods. 2006;18(1):59–82.

[51] King N. Doing template analysis. Qualit Organizat Res: Core Methods Curr Challeng. 2012;(426):426–450.

[52] Braun V, Clarke V. Using thematic analysis in psychology. Qual Res Psychol. 2006;3(2):77–101.

[53] Hofstede G. Cultural differences in teaching and learning. Int J Intercult Relat. 1986;10(3):301–320. doi: 10.1016/0147-1767(86)90015-5

[54] Holland D. Identity and agency in cultural worlds. Arnhem: Harvard University Press; 2001.

[55] Holland D, Lachicotte Jr W, Skinner D, et al. Identity and agency in cultural worlds. Boston, Massachusetts.1998.

[56] Watling C, Driessen E, van der Vleuten CP, et al. Music lessons: revealing medicine's learning culture through a comparison with that of music. Med Educ. 2013;47(8):842–850. 23837431 10.1111/medu.12235

[57] Bing-You R, Ramani S, Ramesh S, et al. The interplay between residency program culture and feedback culture: a cross-sectional study exploring perceptions of residents at three institutions. Med Educ Online. 2019;24(1):1611296. 31038417 10.1080/10872981.2019.1611296 PMC6493320

[58] Ramani S, Post SE, Könings K, et al. “It's just not the culture”: a qualitative study exploring residents' perceptions of the impact of institutional culture on feedback. Teach Learn Med. 2017;29(2):153–161. 28001442 10.1080/10401334.2016.1244014

[59] Ramani S, Könings KD, Mann KV, et al. About politeness, face, and feedback: exploring resident and faculty perceptions of how institutional feedback culture influences feedback practices. Acad Med. 2018;93(9):1348–1358. 29517523 10.1097/ACM.0000000000002193

[60] Watling C, Ginsburg S, LaDonna K, et al. Going against the grain: an exploration of agency in medical learning. Med Educ. 2021;55(8):942–950. 33780013 10.1111/medu.14532

[61] Bates J, Konkin J, Suddards C, et al. Student perceptions of assessment and feedback in longitudinal integrated clerkships. Med Educ. 2013;47(4):362–374. 23488756 10.1111/medu.12087

[62] Sargeant J, Lockyer JM, Mann K, et al. he R2C2 model in residency education: how does it foster coaching and promote feedback use? Acad Med. 2018;93(7):1055–1063. 29342008 10.1097/ACM.0000000000002131

[63] Gee JP. An introduction to discourse analysis: theory and method. New York: Routledge; 2014.

[64] Gaufberg EH, Batalden M, Sands R, et al. The hidden curriculum: what can we learn from third-year medical student narrative reflections? Acad Med. 2010;85(11):1709–1716. 20881818 10.1097/ACM.0b013e3181f57899

[65] Scott KM, Caldwell PH, Barnes EH, et al. “Teaching by humiliation” and mistreatment of medical students in clinical rotations: a pilot study. Med J Australia. 2015;203(4):185–185. 10.5694/mja15.00189 26268289

[66] Gu Q, Schweisfurth M. Who adapts? Beyond cultural models of ‘the’ Chinese learner. Lang Cult Curricul. 2006;19(1):74–89.

[67] Shulman LS. Signature pedagogies in the professions. Daedalus. 2005;134(3):52–59. doi: 10.1162/0011526054622015

[68] Hager P. Theories of workplace learning. In: The SAGE handbook of workplace learning. 2011. p. 17–31.

[69] Molloy E, Boud D, Henderson M. Developing a learning-centred framework for feedback literacy. Assess Eval High Educ. 2020;45(4):527–540.

[70] Dweck CS, Leggett EL. A social-cognitive approach to motivation and personality. Psychol Rev. 1988;95(2):256.

[71] Liao KC, Ajjawi R, Peng CH, et al. Striving to thrive or striving to survive: professional identity constructions of medical trainees in clinical assessment activities. Med Educ. 2023. 10.1111/medu.15152 37394612

[72] Sargeant J, Lockyer J, Mann K, et al. Facilitated reflective performance feedback: developing an evidence-and theory-based model that builds relationship, explores reactions and content, and coaches for performance change (R2C2). Acad Med. 2015;90(12):1698–1706. doi: 10.1097/ACM.0000000000000809 26200584

[73] Sargeant J, Mann K, Sinclair D, et al. Understanding the influence of emotions and reflection upon multi-source feedback acceptance and use. Adv Health Sci Educ. 2008;13(3):275–288. 10.1007/s10459-006-9039-x 17091339

[74] Hofstede G. Culture's consequences: international differences in work-related values (Vol. 5). California: Sage; 1984.

[75] Monrouxe LV, Rees CE. “It’s just a clash of cultures”: emotional talk within medical students’ narratives of professionalism dilemmas. 17. Advances in Health Sciences Education; 2012. p. 671–701. 10.1007/s10459-011-9342-z 22187205

[76] Roudometof V. Glocalization: a critical introduction. Routledge; 2016.

[77] Bennett A. The post-subcultural turn: some reflections 10 years on. J Youth Stud. 2011;14(5):493–506.

[78] Nwalozie CJ. Rethinking subculture and subcultural theory in the study of youth crime–A theoretical discourse. 2015.

[79] Karle H, Christensen L, Gordon D, et al. Neo-colonialism versus sound globalisation policy in medical education. Med Educ. 2008;42(10):956–958. 18823513 10.1111/j.1365-2923.2008.03155.x

[80] Lockyer J, Armson H, Könings KD. In-the-moment feedback and coaching: improving R2C2 for a new context. J Grad Med Educ. 2020;12(1):27–35. 32089791 10.4300/JGME-D-19-00508.1 PMC7012514

